# Integrating CT-Based Lung Fibrosis and MRI-Derived Right Ventricular Function for the Detection of Pulmonary Hypertension in Interstitial Lung Disease

**DOI:** 10.3390/jcm14155329

**Published:** 2025-07-28

**Authors:** Kenichi Ito, Shingo Kato, Naofumi Yasuda, Shungo Sawamura, Kazuki Fukui, Tae Iwasawa, Takashi Ogura, Daisuke Utsunomiya

**Affiliations:** 1 Department of Diagnostic Radiology, Yokohama City University Graduate School of Medicine, Yokohama 236-0004, Japan; kenichiito0909@gmail.com (K.I.); yasuda.nao.mz@yokohama-cu.ac.jp (N.Y.); sawa0808@yokohama-cu.ac.jp (S.S.); d_utsuno@yokohama-cu.ac.jp (D.U.); 2 Department of Cardiology, Kanagawa Cardiovascular and Respiratory Center, Yokohama 236-0051, Japan; fukui.0o400@kanagawa-pho.jp; 3 Department of Diagnostic Radiology, Kanagawa Cardiovascular and Respiratory Center, Yokohama 236-0051, Japan; tae_i_md@wb3.so-net.ne.jp; 4 Department of Respiratory Medicine, Kanagawa Cardiovascular and Respiratory Center, Yokohama 236-0051, Japan; takaoguogu@gmail.com

**Keywords:** interstitial pneumonia, pulmonary fibrosis, computed tomography, right ventricular function, magnetic resonance imaging

## Abstract

**Background/Objectives**: Interstitial lung disease (ILD) is frequently complicated by pulmonary hypertension (PH), which is associated with reduced exercise capacity and poor prognosis. Early and accurate non-invasive detection of PH remains a clinical challenge. This study evaluated whether combining quantitative CT analysis of lung fibrosis with cardiac MRI-derived measures of right ventricular (RV) function improves the diagnostic accuracy of PH in patients with ILD. **Methods**: We retrospectively analyzed 72 ILD patients who underwent chest CT, cardiac MRI, and right heart catheterization (RHC). Lung fibrosis was quantified using a Gaussian Histogram Normalized Correlation (GHNC) software that computed the proportions of diseased lung, ground-glass opacity (GGO), honeycombing, reticulation, consolidation, and emphysema. MRI was used to assess RV end-systolic volume (RVESV), ejection fraction, and RV longitudinal strain. PH was defined as a mean pulmonary arterial pressure (mPAP) ≥ 20 mmHg and pulmonary vascular resistance ≥ 3 Wood units on RHC. **Results**: Compared to patients without PH, those with PH (*n* = 21) showed significantly reduced RV strain (−13.4 ± 5.1% vs. −16.4 ± 5.2%, *p* = 0.026) and elevated RVESV (74.2 ± 18.3 mL vs. 59.5 ± 14.2 mL, *p* = 0.003). CT-derived indices also differed significantly: diseased lung area (56.4 ± 17.2% vs. 38.4 ± 12.5%, *p* < 0.001), GGO (11.8 ± 3.6% vs. 8.65 ± 4.3%, *p* = 0.005), and honeycombing (17.7 ± 4.9% vs. 12.8 ± 6.4%, *p* = 0.0027) were all more prominent in the PH group. In receiver operating characteristic curve analysis, diseased lung area demonstrated an area under the curve of 0.778 for detecting PH. This increased to 0.847 with the addition of RVESV, and further to 0.854 when RV strain was included. Combined models showed significant improvement in risk reclassification: net reclassification improvement was 0.700 (*p* = 0.002) with RVESV and 0.684 (*p* = 0.004) with RV strain; corresponding IDI values were 0.0887 (*p* = 0.03) and 0.1222 (*p* = 0.01), respectively. **Conclusions**: Combining CT-based fibrosis quantification with cardiac MRI-derived RV functional assessment enhances the non-invasive diagnosis of PH in ILD patients. This integrated imaging approach significantly improves diagnostic precision and may facilitate earlier, more targeted interventions in the management of ILD-associated PH.

## 1. Introduction

Interstitial lung disease (ILD) comprises a heterogeneous group of disorders characterized by progressive pulmonary fibrosis, which significantly impairs respiratory function, reduces quality of life, and worsens overall prognosis [1,2,3]. A common and serious complication of ILD is the development of pulmonary hypertension (PH), a condition marked by elevated pulmonary arterial pressures that further deteriorates clinical outcomes [4,5,6]. Early and accurate detection of PH in patients with ILD is essential for guiding therapeutic decisions and improving patient outcomes. Although right heart catheterization (RHC) remains the gold standard for diagnosing PH, it is invasive and carries procedural risks, limiting its widespread applicability in clinical practice.

Recent advancements in imaging technologies have facilitated non-invasive assessment of both pulmonary fibrosis and right ventricular (RV) dysfunction—key components in the pathophysiology of PH. Computed tomography (CT) enables quantitative evaluation of lung parenchymal abnormalities, including fibrotic changes [7,8,9,10]. Notably, the Gaussian Histogram Normalized Correlation (GHNC) segmentation system has been developed to classify ILD patterns by analyzing pixel-wise histogram correlations with representative disease templates. This technique allows objective quantification of fibrotic burden and has demonstrated prognostic significance, with higher fibrotic scores associated with worse survival [9]. In parallel, cardiac magnetic resonance imaging (MRI) provides a detailed assessment of RV function. Beyond conventional metrics such as volumes and ejection fraction, feature-tracking MRI enables measurement of RV longitudinal strain, a sensitive marker of early dysfunction. The presence of PH has been associated with impaired RV strain, which in turn has been shown to predict short-term mortality in patients with ILD [11]. Thus, RV strain may serve as a useful non-invasive prognostic marker in this population.

Given the complementary strengths of CT in evaluating pulmonary fibrosis and MRI in assessing RV performance, an integrated imaging approach may offer a robust, non-invasive strategy for the early detection and prognostication of PH in patients with ILD. This study aims to evaluate the diagnostic accuracy of a novel approach for diagnosing pulmonary hypertension, which combines CT-based quantification of fibrosis and MRI-based analysis of right ventricular (RV) strain, using RHC as the reference standard.

## 2. Materials and Methods

### 2.1. Study Subjects

This retrospective study was approved by the institutional review board, and the requirement for informed consent was waived. A total of 72 patients with ILD and suspected pulmonary hypertension (PH) who underwent RHC between 2011 and 2020 were retrospectively reviewed. The diagnosis of ILD was established according to the criteria of the American Thoracic Society and European Respiratory Society through a multidisciplinary discussion involving pulmonologists, radiologists, and pathologists [12]. All patients demonstrated progressive fibrotic changes on chest computed tomography (CT) scans. Among the 72 patients, 35 (49%) underwent surgical lung biopsy, which histopathologically confirmed pulmonary fibrosis, including usual interstitial pneumonia (UIP) pattern (*n* = 14), nonspecific interstitial pneumonia (NSIP) pattern (*n* = 12), unclassifiable fibrosis (*n* = 8), and desquamative interstitial pneumonia (DIP) pattern (*n* = 1). Exclusion criteria included acute exacerbation of ILD, significant pleural effusion, pneumothorax, known cardiomyopathies (e.g., hypertrophic cardiomyopathy, dilated cardiomyopathy, cardiac sarcoidosis, cardiac amyloidosis), moderate to severe valvular heart disease, pulmonary vascular disease (e.g., chronic thromboembolic pulmonary hypertension), and pulmonary capillary wedge pressure > 15 mmHg on RHC. PH was defined as a mean pulmonary arterial pressure (mPAP) ≥ 20 mmHg at rest and a pulmonary vascular resistance (PVR) ≥ 3 Wood units, as determined by RHC [13]. Lung volumes (vital capacity [VC], forced expiratory volume in 1 s [FEV_1_], total lung capacity [TLC]) and diffusing capacity of the lungs were measured using standard techniques with the following equipment: CHESTAC-8800 and CHESTAC-33 (Chest MI), and FUDAC-77 (Fukuda Denshi). Results were expressed as a percentage of predicted values, based on established reference standards [10]. This retrospective study was approved by the Institutional Review Board of Kanagawa Cardiovascular and Respiratory Center (Ethical code: KCRC-20-1003, approved on 20 April 2020).

### 2.2. CT Image Acquisition and Analysis

**Figure 1:**
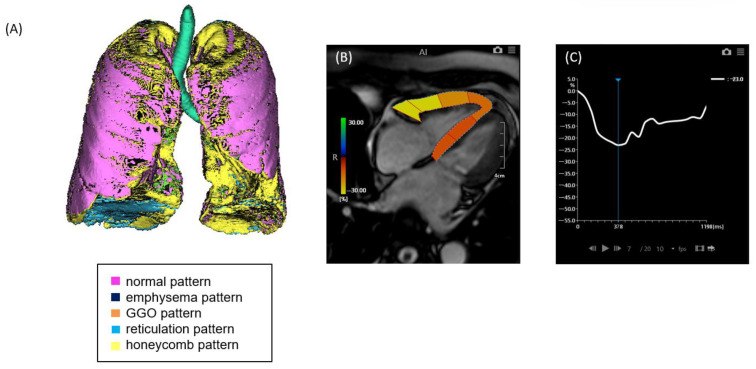
A case of ILD with mild pulmonary fibrosis and preserved right ventricular (RV) strain. (**A**) Quantitative CT-based 3D reconstruction of lung parenchymal patterns. Normal lung parenchyma (pink), emphysema (dark blue), ground-glass opacity (GGO, orange), reticulation (light blue), and honeycombing (yellow) are segmented. (**B**) Cardiac MRI showing right ventricular strain analysis using feature tracking. (**C**) Strain–time curve of the right ventricle showing preserved peak longitudinal strain (−23.0%).

**Figure 2:**
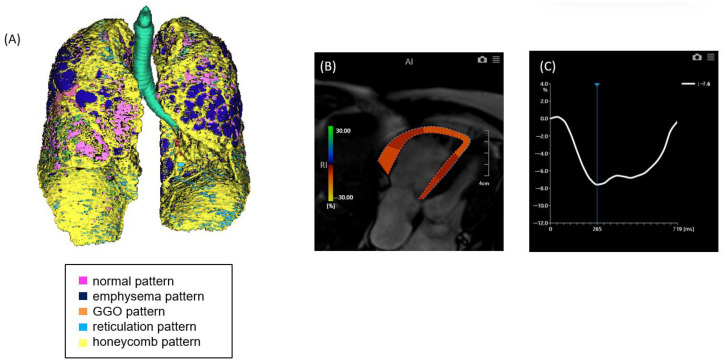
A case of ILD with advanced pulmonary fibrosis and impaired right ventricular (RV) strain. (**A**) Quantitative CT-based 3D reconstruction of lung parenchymal patterns. Normal lung parenchyma (pink), emphysema (dark blue), ground-glass opacity (GGO, orange), reticulation (light blue), and honeycombing (yellow) are segmented, with extensive honeycombing and emphysema observed. (**B**) Cardiac MRI showing RV strain analysis with feature tracking, indicating reduced myocardial deformation predominantly in the free wall. (**C**) Strain–time curve of the right ventricle showing severely reduced peak longitudinal strain (−7.6%).

Non-contrast chest computed tomography (CT) scans were obtained during full inspiration in the supine position using multidetector CT scanners (Aquilion 64, Aquilion ONE 320, or Aquilion Precision 160; Canon Medical Systems, Japan). The slice thickness was 0.5 or 1.0 mm. The median interval between CT and RHC was 71 days (mean, 88.5 days; range, 0–325 days). CT data were analyzed using the GHNC system, which classifies lung parenchyma into six categories—normal (N), emphysema (E), ground-glass opacity (GGO, G), consolidation (C), reticulation (R), and honeycombing (H)—based on local histogram features and CT attenuation values [9,10]. These categories were predefined using datasets from healthy individuals and patients not included in the current study. The system automatically calculated the volume of each category and the total CT lung volume (CTLV), displaying the results as color-coded overlays. Volumetric data for each category were expressed as percentages of the CTLV. The diseased lung area was defined as the sum of all non-normal parenchymal patterns, including GGO, honeycombing, reticulation, emphysema, and consolidation (Figure 1 and Figure 2). In this study, the extent of fibrosis was quantitatively assessed using the percentage of diseased lung area—defined as the sum of GGO, reticulation, honeycombing, consolidation, and emphysema—relative to the total CT lung volume (i.e., expressed as a percentage of the entire lung). This quantitative measure was used as a surrogate marker for the stage of pulmonary fibrosis.

### 2.3. MRI Image Acquisition and Analysis

Cardiac MRI was performed using a 1.5-T scanner and 32-channel cardiac coils (Achieva, Philips Healthcare, Best, The Netherlands). Cine images of the left ventricle (LV) were acquired in two-chamber, four-chamber, and short-axis orientations using a steady-state free precession (SSFP) sequence with the following parameters: repetition time (TR), 4.1 ms; echo time (TE), 1.7 ms; flip angle, 55°; field of view, 350 × 350 mm^2^; acquisition matrix, 128 × 128; and 20 phases per cardiac cycle. The median interval between cardiac MRI and RHC was 20 days (mean, 37.2 days; range, 20–366 days), and right ventricular (RV) volumes and ejection fraction (RVEF) were calculated from four-chamber cine image stacks encompassing the entire RV. The RV endocardial borders were manually traced at end-diastole and end-systole, excluding trabeculae. RV longitudinal strain was assessed using feature-tracking analysis software (Vitrea Version 7.8.0, Canon Medical Systems, Otawara, Tochigi, Japan). The epicardial and endocardial borders of the RV were manually delineated on four-chamber cine images, and the peak of the strain curve was recorded as the RV longitudinal strain value.

### 2.4. Right Heart Catheterization

All patients underwent RHC for the definitive diagnosis and hemodynamic characterization of PH. The procedure was performed using a standard thermodilution Swan-Ganz catheter (ControlCath, Edwards Lifesciences, Irvine, CA, USA), which was inserted via the right internal jugular vein under fluoroscopic guidance. Hemodynamic parameters measured included mPAP, systolic and diastolic pulmonary artery pressures, right atrial pressure (RAP), right ventricular pressure, and pulmonary capillary wedge pressure (PCWP). Cardiac output (CO) was determined using either the thermodilution technique or, when necessary, the Fick principle—depending on the stability of the patient’s respiratory status and oxygen consumption measurements. The choice of method was based on the clinical discretion of the attending cardiologist or pulmonologist performing the procedure. Pulmonary vascular resistance (PVR) was subsequently calculated using the following formula:

*PVR* = *mean PAP* − *PCWP* / *CO* (*dyne · 
sec · cm* − *5*).

PH was defined hemodynamically as a mPAP ≥ 20 mmHg at rest and a PVR ≥ 3 Wood units, consistent with current international guidelines.

### 2.5. Statistical Analysis

Subjects were categorized into two groups based on the presence or absence of PH. Continuous variables were expressed as mean ± standard deviation (SD), and comparisons between the two groups were performed using the Mann–Whitney U test. To assess the independent predictive value of CT- and MRI-derived parameters for the presence of PH, multivariate logistic regression analysis was conducted. Receiver operating characteristic (ROC) curve analysis was used to evaluate and compare the diagnostic performance of CT and MRI measurements in detecting PH. The area under the curve (AUC) was calculated for each parameter. To determine whether combining CT and MRI parameters provided incremental diagnostic value, we performed step-wise model-based analysis. The added value of each parameter was further evaluated using net reclassification improvement (NRI) and integrated discrimination improvement (IDI) indices. A *p*-value of <0.05 was considered statistically significant. All statistical analyses were performed using SPSS software (version 21.0; IBM Corp., Armonk, NY, USA) and R software (version 3.0.2; The R Foundation for Statistical Computing, Vienna, Austria).

## 3. Results

### 3.1. Patient Characteristics

A total of 72 patients with ILD were included in this study. Among them, 21 patients were diagnosed with pulmonary hypertension (PH) based on right heart catheterization. The baseline characteristics of patients with and without PH are summarized in Table 1. The average age of the entire cohort was 70.5 ± 7.4 years, with no significant age difference between PH and non-PH groups (71.9 ± 6.1 vs. 70.0 ± 8.0 years, *p* = 0.27). The majority of patients were male (61%), with similar sex distribution between groups. Patients with PH had significantly higher dyspnea severity, as reflected by the MRC dyspnea scale (2.8 ± 0.9 vs. 1.5 ± 0.9, *p* < 0.001). Diastolic blood pressure was slightly higher in the PH group (81 ± 9.8 mmHg vs. 76 ± 9.0 mmHg, *p* = 0.033), while systolic pressure and heart rate were not statistically different. Brain natriuretic peptide (BNP) levels were markedly elevated in the PH group (129 ± 211 pg/mL vs. 30 ± 23 pg/mL, *p* = 0.044), consistent with increased right ventricular strain. Pulmonary function tests revealed significantly reduced diffusing capacity (DL_CO_) in PH patients (47 ± 17% vs. 71 ± 22% predicted, *p* < 0.001), along with lower forced vital capacity (FVC) (72 ± 15% vs. 85 ± 25%, *p* = 0.01) and forced expiratory volume in one second (FEV1, %) (70 ± 19% vs. 81 ± 20%, *p* = 0.037). Other variables, including body mass index, serum KL-6, CRP, LDH, and creatinine levels, did not differ significantly between groups.

### 3.2. Comparison of Chest CT and Cardiac MRI Parameters of ILD with and Without PH

**Figure 3:**
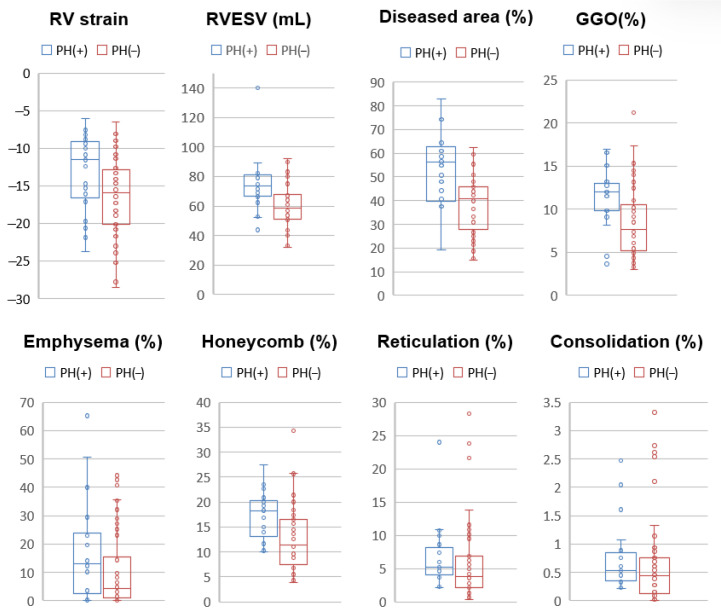
Boxplots of CT and MRI parameters comparing patients with and without pulmonary hypertension (PH). Patients with PH [PH(+), blue] demonstrated significantly impaired right ventricular (RV) function, as indicated by lower RV longitudinal strain (*p* = 0.026) and higher RV end-systolic volume (RVESV, *p* = 0.003) compared to those without PH [PH(−), red]. On CT analysis, PH(+) patients exhibited significantly greater diseased lung area (*p* < 0.001), ground-glass opacity (GGO, *p* = 0.005), and honeycombing (*p* = 0.0027). No significant differences were observed for emphysema (*p* = 0.12), reticulation (*p* = 0.23), or consolidation (*p* = 0.27).

Cardiac MRI and chest CT findings were compared between patients with and without pulmonary hypertension (PH), as summarized in Table 2. Patients with PH demonstrated significantly impaired right ventricular (RV) function. RV longitudinal strain was lower in the PH group (−13.4 ± 5.1%) compared to the non-PH group (−16.4 ± 5.2%, *p* = 0.026). In addition, RV end-systolic volume (RVESV) was significantly higher in patients with PH (74.2 ± 18.3 mL vs. 59.5 ± 14.2 mL, *p* = 0.003), suggesting reduced RV contractile performance. Quantitative CT analysis revealed a significantly greater extent of lung involvement in patients with PH. The proportion of diseased lung area was substantially higher in the PH group (56.4 ± 17.2%) compared to those without PH (38.4 ± 12.5%, *p* < 0.001). Among specific parenchymal patterns, GGO was more prominent in PH patients (11.8 ± 3.6% vs. 8.65 ± 4.3%, *p* = 0.005), as was honeycombing (17.7 ± 4.9% vs. 12.8 ± 6.4%, *p* = 0.0027). Other CT-derived parameters—including emphysema, reticulation, and consolidation—did not differ significantly between the two groups (Figure 3).

### 3.3. Diagnostic Performance of CT and MRI Parameters

**Figure 4:**
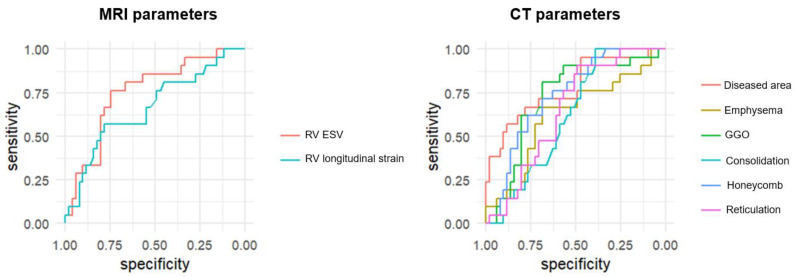
Receiver operating characteristic (ROC) curves of CT and MRI parameters for the diagnosis of pulmonary hypertension (PH). ROC curves compare the diagnostic performance of cardiac MRI-derived parameters (RV strain, RV end-systolic volume [RVESV]) and CT-derived parameters (diseased lung area, ground-glass opacity [GGO], honeycombing, reticulation, consolidation, and emphysema) for detecting PH in patients with interstitial lung disease. The area under the curve (AUC) was highest for diseased lung area (AUC = 0.778), followed by RVESV (AUC = 0.754), honeycombing (AUC = 0.738), and GGO (AUC = 0.724). RV strain showed moderate diagnostic utility (AUC = 0.665), while emphysema, reticulation, and consolidation showed lower diagnostic value.

**Figure 5:**
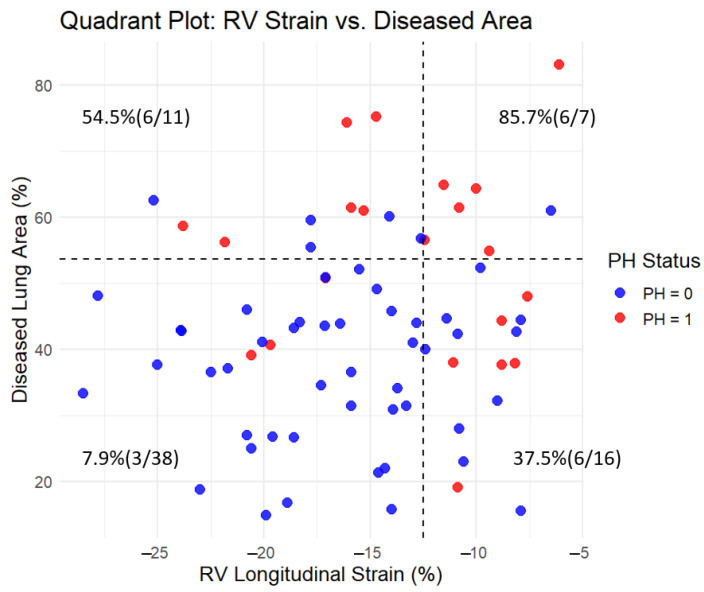
Quadrant plot of RV longitudinal strain and diseased lung area with pulmonary hypertension (PH) prevalence. The plot divides the cohort into four quadrants based on threshold values determined by ROC analysis using the Youden index: −12.5% for RV longitudinal strain (*x*-axis) and 53.6% for diseased lung area (*y*-axis). Each dot represents a subject, color-coded by PH status (red = PH present, blue = PH absent). Dashed lines indicate the threshold values. In each quadrant, the percentage and count of patients with PH (n/N) are shown. The prevalence of PH was highest in the upper right quadrant (85.7%, 6/7) and lowest in the lower left quadrant (7.9%, 3/38), demonstrating the combined impact of reduced RV strain and increased lung fibrosis on PH presence.

The diagnostic performance of individual CT and MRI parameters for detecting PH is summarized in Table 3. Among MRI-derived measurements, RVESV showed the highest diagnostic accuracy with an AUC of 0.754 (95% CI: 0.631–0.876, *p* < 0.001), followed by RV longitudinal strain with an AUC of 0.665 (95% CI: 0.522–0.808, *p* = 0.022). Among CT-derived parameters, the proportion of diseased lung area demonstrated the strongest diagnostic performance (AUC: 0.778, 95% CI: 0.685–0.921, *p* < 0.001). Honeycombing (AUC: 0.738, *p* < 0.001) and GGO (AUC: 0.724, *p* = 0.001) also showed significant association with PH. Other CT features, including emphysema and consolidation, exhibited lower diagnostic value with non-significant *p*-values (Figure 4). In addition, a quadrant analysis using ROC-based thresholds (−12.5% for RV longitudinal strain and 53.6% for diseased lung area) was performed to stratify patients. The prevalence of PH in the upper right quadrant (i.e., patients with both impaired RV strain and greater extent of lung fibrosis) was 85.7% (6/7), whereas it was only 7.9% (3/38) in the lower left quadrant (i.e., patients with preserved RV strain and limited fibrosis) (Figure 5).

### 3.4. Incremental Value of Combining CT and MRI Parameters

To evaluate whether combining CT and MRI parameters improves PH detection, we performed model-based analysis using ROC metrics and reclassification indices (Table 4). The model based on CT-derived diseased area alone yielded an AUC of 0.778. Adding RVESV improved the AUC to 0.847, and further inclusion of RV strain increased it to 0.860. This step-wise improvement was supported by significant net reclassification improvement (NRI: 0.700 and 0.6835, respectively; *p* = 0.002 and *p* = 0.004) and integrated discrimination improvement (IDI: 0.0887 and 0.1222; *p* = 0.03 and *p* = 0.01, respectively). These findings collectively indicate that combining functional cardiac MRI data with quantitative CT analysis substantially improves diagnostic discrimination and clinical stratification for detecting PH in patients with ILD.

## 4. Discussion

This study demonstrates that combining CT-based quantitative assessment of lung fibrosis with cardiac MRI-derived measures of right ventricular function enhances the non-invasive diagnosis of pulmonary hypertension in patients with interstitial lung disease. Given the limitations and risks of right heart catheterization, there is an urgent need for accurate, reproducible, and patient-friendly imaging alternatives in this vulnerable population.

Pulmonary hypertension is a common and serious complication in ILD, associated with worsened symptoms, reduced exercise capacity, and poor prognosis [13,14]. PH occurs in 8–15% of early ILD cases, rising to 30–50% in advanced stages and exceeding 60% in end-stage disease [6]. However, the prevalence of PH in ILD is likely underestimated, in part due to the low uptake of RHC in routine clinical practice. Non-invasive imaging modalities such as echocardiography, though widely accessible, are often suboptimal in ILD patients due to poor acoustic windows and interobserver variability [15]. In contrast, CT and cardiac MRI offer high spatial and temporal resolution, quantitative capabilities, and reproducibility. In this study, we found that the CT-derived proportion of diseased area alone yielded a strong diagnostic performance (AUC = 0.778) in identifying PH. This was further enhanced by adding MRI-derived RVESV, and further still with RV longitudinal strain (AUC = 0.879). The incremental improvement in diagnostic power was supported by significant NRI and IDI metrics. These findings underscore the complementary nature of pulmonary parenchymal and right heart functional imaging in assessing ILD-related vascular disease.

From a pathophysiological perspective, PH in ILD is driven by fibrotic destruction of the pulmonary vascular bed, hypoxic vasoconstriction, and vascular remodeling, which together increase pulmonary vascular resistance [16,17]. These hemodynamic changes impose a pressure overload on the RV, ultimately leading to RV remodeling and dysfunction. RV strain, as derived from feature-tracking cardiac MRI, serves as an early and sensitive marker of this dysfunction—often preceding changes in ejection fraction or chamber size [18,19]. The ability to detect these subclinical changes is particularly valuable in ILD, where PH progression can be insidious and symptoms often overlap with underlying lung disease. In parallel, the GHNC system used for CT analysis allows for pixel-based classification of parenchymal changes, distinguishing normal lung, ground-glass opacity, consolidation, reticulation, emphysema, and honeycombing [8]. Quantitative CT analysis using the GHNC system enables automated classification of parenchymal patterns, such as ground-glass opacity, honeycombing, consolidation, and emphysema. This method constructs reference histograms from both original and differential images of representative samples for each abnormality [10,20]. For every pixel in the target CT image, a local Gaussian histogram is generated based on a 50-pixel neighborhood weighted by a Gaussian kernel. The resulting pixel-wise histogram is then compared with the reference histograms using normalized correlation, and the pixel is assigned to the abnormality class with the highest similarity. This approach allows for objective, reproducible segmentation of complex and heterogeneous lung pathology. Our findings are consistent with previous reports suggesting that the extent of preserved (i.e., normal) lung parenchyma may be inversely associated with PH in ILD. For example, in a study of 40 patients with chronic fibrosing idiopathic interstitial pneumonia, a computer-aided 3D CT analysis revealed that the percentage of normal lung was significantly correlated with mean pulmonary artery pressure measured by RHC. The proportion of normal lung was a significant predictor of PH (odds ratio, 0.92; *p* = 0.02), and showed good discriminative ability with an AUC of 0.849. In contrast, the relative volumes of individual abnormal patterns, such as emphysema or fibrosis, had limited predictive value [21]. These results support the concept that preserved lung volume, rather than the extent of specific lesions, may serve as a more robust imaging biomarker of PH in fibrotic lung diseases. Furthermore, in a subsequent study, the proportion of subpleural honeycombing identified by GHNC analysis was found to be an independent predictor of mortality, with patients exhibiting ≥ 5% honeycombing showing significantly reduced long-term survival [9]. These findings support the role of GHNC-based fibrosis quantification as a non-invasive imaging biomarker with both prognostic and pathophysiological relevance. Our current study builds on prior evidence by demonstrating that specific CT patterns—particularly ground-glass opacity and honeycombing—are more prevalent in ILD patients who have developed pulmonary hypertension. These findings support the concept that progressive parenchymal lung disease contributes to the development of PH and highlight the potential of GHNC-derived metrics, not only for staging and prognosis of ILD, but also for the non-invasive identification of associated vascular complications.

Beyond diagnosis and monitoring of PH, this imaging-based strategy may also serve as a predictive biomarker for treatment response in patients receiving antifibrotic therapies. Pirfenidone and nintedanib, approved based on large-scale randomized trials such as ASCEND and INPULSIS, have demonstrated efficacy in slowing disease progression in idiopathic pulmonary fibrosis (IPF) and progressive fibrosing ILDs [22,23]. However, identifying patients who will derive the most benefit from antifibrotic therapy remains a major clinical challenge. Quantitative imaging biomarkers—such as fibrotic lesion proportion on CT and early signs of RV strain on cardiac MRI—may provide insight into individual disease trajectories and treatment responsiveness. For example, a patient with extensive subpleural honeycombing but preserved RV strain may represent an earlier disease stage that is more amenable to antifibrotic intervention. Conversely, evidence of progressive RV dysfunction despite limited fibrotic change may suggest the need for additional therapies targeting pulmonary vascular remodeling. As such, these imaging parameters may facilitate early, personalized therapeutic decision-making, guiding both initiation and escalation of antifibrotic treatment. Future studies should explore the role of these imaging markers in predicting therapeutic outcomes, particularly in prospective settings where serial CT and MRI evaluations can be correlated with longitudinal changes in pulmonary function, exercise capacity, and survival. Integration with serum biomarkers and pulmonary function testing may further enhance predictive accuracy, supporting the development of a multi-modal precision medicine framework in ILD.

### Limitations

Several limitations should be acknowledged in this study. This study was retrospective and conducted at a single institution, which may limit external generalizability. The number of patients with confirmed PH was relatively small, potentially affecting statistical power. Additionally, the time interval between imaging and RHC varied, although efforts were made to ensure temporal proximity. Interobserver variability in manual segmentation, particularly for MRI-derived strain analysis, was not formally assessed and should be addressed in future studies. Lastly, this study focused on diagnosis rather than prognosis; future longitudinal studies are warranted to determine whether these imaging parameters can predict clinical outcomes such as mortality or functional decline. Furthermore, while our imaging-based approach provides useful non-invasive insights, it cannot replace RHC, which remains the gold standard for diagnosing PH. Rather, it may help identify patients who warrant further invasive evaluation. We also acknowledge that a PVR threshold of >3 WU was used based on earlier guidelines, whereas the 2022 ESC/ERS definition adopts a lower threshold of >2 WU. This discrepancy may have resulted in misclassification and should be recognized as a significant limitation. In assessing RV function, we used global RV longitudinal strain instead of RV free wall strain due to software limitations and challenges in isolating the free wall in patients with abnormal anatomy. Given that RV free wall strain may more accurately reflect RV function, this methodological choice may have influenced our results. Finally, other RV parameters recommended in clinical guidelines, such as RVEF and SVI, were not included in this analysis and warrant consideration in future investigations. It remains controversial whether every patient with PH secondary to lung disease requires RHC. Data on the use of PAH-targeted therapies in this population are limited, with inhaled treprostinil being the only agent cautiously recommended, and even then, as a class IIb indication. Thus, non-invasive imaging approaches that aid in patient selection for invasive evaluation may hold clinical value and deserve further exploration.

## 5. Conclusions

In conclusion, the integration of CT-based lung fibrosis assessment and MRI-derived right ventricular function significantly improves the non-invasive diagnosis of pulmonary hypertension in interstitial lung disease. The diagnostic accuracy increased from an AUC of 0.778 using lung fibrosis alone to 0.854 when combined with RVESV and RV strain. These combined models also significantly enhanced risk reclassification, supporting their potential to enable earlier and more accurate clinical management.

## Figures and Tables

**Table 1:** Patients’ characteristics.

	All Patients (*n* = 72)	Patients with PH (*n* = 21)	Patients Without PH (*n* = 51)	* *p*-Value
Age	70.5 ± 7.4	71.9 ± 6.1	70.0 ± 8.0	0.27
Sex (Male)	44 (61%)	14 (67%)	30 (59%)	
MRC dyspnea scale	1.9 ± 1.1	2.8 ± 0.9	1.5 ± 0.9	<0.001
BMI (kg/m^2^)	23.0 ± 3.1	23.2 ± 2.9	23.0 ± 3.2	0.77
Systolic blood pressure (mmHg)	136 ± 17	138 ± 15	135 ± 18	0.44
Diastolic blood pressure (mmHg)	77 ± 9.5	81 ± 9.8	76 ± 9.0	0.033
Heart rate (bpm)	74 ± 14	80 ± 16	72 ± 12	0.06
BNP (pg/mL)	59 ± 123	129 ± 211	30 ± 23	0.044
Creatinine (mg/dL)	1.0 ± 1.2	1.4 ± 2.2	0.8 ± 0.2	0.309
KL-6 (U/mL)	1025 ± 645	1057 ± 572	1011 ± 677	0.773
LDH (IU/L)	244 ± 54	249 ± 59	242 ± 52	0.645
CRP (mg/dL)	0.39 ± 0.55	0.51 ± 0.65	0.34 ± 0.50	0.296
FVC, % predicted	82 ± 23	72 ± 15	85 ± 25	0.01
FEV1, % predicted	78 ± 20	70 ± 19	81 ± 20	0.037
DLco, % predicted	65 ± 23	47 ± 17	71 ± 22	<0.001

* *p* value represents the significance of difference between patients with PH and those without.

**Table 2:** Comparison of CT and MRI parameters between patients with and without PH.

Modality	Parameter	PH Present (*n* = 21)	PH Absent (*n* = 51)	* *p*-Value
MRI (Heart)	RV strain	−13.4 ± 5.1	−16.4 ± 5.2	0.026
	RVESV	74.2 ± 18.3	59.5 ± 14.2	0.003
CT (Lung)	Diseased area	56.4 ± 17.2	38.4 ± 12.5	<0.001
	GGO	11.8 ± 3.6	8.65 ± 4.3	0.005
	Emphysema	17.4 ± 17.2	10.6 ± 12.9	0.12
	Honeycomb	17.7 ± 4.9	12.8 ± 6.4	0.0027
	Reticulation	8.10 ± 8.04	5.66 ± 5.75	0.23
	Consolidation	1.48 ± 3.20	0.662 ± 0.798	0.27

* *p* value represents the significance of difference between patients with PH and those without.

**Table 3:** Diagnostic performance of MRI and CT findings for detecting PH.

Modality	Parameter	AUC	95% CI	*p*-Value	Threshold	Sensitivity, %	Specificity, %
MRI (Heart)	RV strain	0.665	0.522–0.808	0.022	−12.5	57.1	78.4
	RVESV	0.754	0.631–0.876	<0.001	67.2	76.2	74.5
CT (Lung)	Diseased area	0.778	0.685–0.921	<0.001	53.6	57.1	88.2
	Emphysema	0.622	0.451–0.755	0.101	9.93	66.7	68.6
	GGO	0.724	0.610–0.864	0.001	9.77	81.0	68.6
	Consolidation	0.622	0.526–0.775	0.058	0.21	100	39.2
	Honeycomb	0.738	0.634–0.864	<0.001	14.8	71.4	68.6
	Reticulation	0.654	0.536–0.788	0.017	3.68	90.5	49.0
MRI + CT	Diseased area + RVESV	0.846	0.748–0.944	<0.001	0.186	90.5	68.6
	Diseased area + RVESV + RV strain	0.860	0.767–0.953	<0.001	0.361	76.2	84.3

AUC: area under the curve; CI: confidence interval; GGO: ground glass opacity; PH: pulmonary hypertension; RV: right ventricle; RVESV: right ventricular end-systolic volume.

**Table 4:** Improvement in diagnostic models using RVESV and RV strain.

Improvement in Discrimination—AUC and NRI				
Model	AUC	NRI (Continuous)	95% CI	*p*-Value
Diseased area only	0.778			
Diseased area + RVESV	0.847	0.700	0.247–1.154	0.002
Diseased area + RVESV + RV strain	0.854	0.6835	0.2147–1.1523	0.004
**Improvement in Reclassification—AUC and IDI**				
**Model**	**AUC**	**NRI (Continuous)**	**95% CI**	***p*** **-value**
Diseased area only	0.778			
Diseased area + RVESV	0.847	0.0887	0.0097–0.1678	0.03
Diseased area + RVESV + RV strain	0.854	0.1222	0.0286–0.2158	0.01

AUC: area under the curve; CI: confidence interval; IDI: integrated discrimination improvement; NRI: net reclassification improvement; RV: right ventricle; RVESV: right ventricular end-systolic volume.

## Data Availability

The datasets used and/or analyzed during the current study are available from the corresponding author on reasonable request.
